# A case report of rapid diagnosis of *Sporothrix globosa* infection using MetaCAP

**DOI:** 10.3389/fmed.2025.1644400

**Published:** 2025-08-11

**Authors:** Cuili Li, Xi Zhong, Shuxian Guo, Feifei Liu, Yanhui Song, Zhiying Zhao, Guan Jiang

**Affiliations:** ^1^Department of Infectious Diseases, KingMed Diagnostics, Guangzhou, Guangdong, China; ^2^Department of Dermatology, Affiliated Hospital of Xuzhou Medical University, Xuzhou, Jiangsu, China

**Keywords:** sporotrichosis, *Sporothrix globosa*, MetaCAP, mNGS, itraconazole

## Abstract

**Background:**

*Sporothrix globosa* (*S. globosa*) is a significant pathogenic fungus responsible for, causing sporotrichosis. Metagenomics capture (MetaCAP), a high-throughput sequencing technology for pathogen nucleic acid detection based on probe capture, facilitates early diagnosis of *S. globosa* infections. Herein, we present a case of *S. globosa* infection diagnosed using MetaCAP.

**Case summary:**

A 47 year-old female, initially diagnosed with reactive perforating collagenosis, developed epidermal erosion at the affected site after self-applying a poorly air-permeable topical patch purchased online. She subsequently exhibited progressive redness, edema, severe pruritus, and an increase in papules that coalesced into plaques following exposure to decaying wood. She revisited our hospital for further consultation. The diagnosis of sporotrichosis caused by *S. globosa* was confirmed through a pathological examination of the affected skin tissue and MetaCAP testing. Then, she was treated with itraconazole and naftifine hydrochloride and ketoconazole cream. After a three-month follow-up, the patient’s skin rash showed significant improvement.

**Conclusion:**

A case of *S. globosa* infection was promptly diagnosed through MetaCAP and effectively treated with itraconazole.

## 1 Introduction

Sporotrichosis is a subacute or chronic infection caused by the *Sporothrix schenckii complex*, a dimorphic fungus ubiquitous in the environment (1). *Sporothrix globosa (S. globosa)* is one of the primary pathogens responsible for sporotrichosis, widely distributed globally but more prevalent in Asian countries such as China, India, and Japan, particularly in northeastern China (2–5). *S. globosa* is a dimorphic fungus characterized by its filamentous form at room temperature (25°C) and its transition to the yeast phase within the body or at 37°C (1, 6). Transmission of *S. globosa* primarily occurs through saprophytic means, where environmental contaminants (such as soil, wood, plants, etc.) cause colonization of the fungus through wounds, commonly seen in individuals with a history of trauma, especially on exposed areas like the extremities and head-and-face region (1, 7, 8). Based on clinical manifestations, sporotrichosis is classified into fixed cutaneous, lymphocutaneous, disseminated cutaneous, and extracutaneous forms. *S. globosa* primarily affects the skin but can also involve mucous membranes, subcutaneous tissues, and adjacent lymphatic vessels. In severe cases, it can disseminate through the blood and lymphatic system, causing systemic damage and posing a life-threatening risk (6, 9). Prompt diagnosis and treatment of *S. globosa* infections are crucial to prevent disease progression.

Metagenomics capture (MetaCAP) is a high-throughput sequencing technology for pathogen nucleic acid detection based on probe capture, which allows for direct detection and analysis of the genetic material (DNA and RNA) of all microorganisms in various types of clinical samples. Inspired by the research conducted by the team of Swedish scientist Svante Pääbo on ancient human genomes using probe capture technology (10, 11), KingMed Diagnostics has integrated probe capture technology with next-generation sequencing technology to independently develop MetaCAP, which features a unique technology characterized by “depleting host genes + million-probe capture.” MetaCAP integrates the priority levels of pathogen concern and pathogen characteristics to design specific million-capture probes that are primarily enriched for over 3,000 pathogen species. These probes hybridize with the target genomic DNA library in the sample that contains the target regions. Simultaneously, in combination with differential depleting host genes technology, it concurrently enriches the pathogen nucleic acid sequence in the samples. After capture, high-throughput sequencing is performed using next-generation sequencing platforms. Complementary bioinformatics software is used to filter, analyze, and interpret the sequencing data, determining the types of potential pathogenic microorganisms, drug resistance, and virulence information present in the tested samples. Currently, MetaCAP has been widely applied in the etiological diagnosis of various clinical infectious diseases, including infections of the central nervous system (12), bloodstream infections (13), septic arthritis (14), and more. It plays a crucial role in diagnosing emerging, mutated, and rare pathogens, as well as mixed and difficult-to-diagnose infections (12–15), thereby aiding in the early detection of non-specific infections caused by *S. globosa*. It significantly shortens the diagnosis time and provides crucial information for the diagnosis and treatment of the disease (13). Here, we report a case of rapid diagnosis of *S. globosa* infection utilizing MetaCAP.

## 2 Case presentation

**FIGURE 1:**
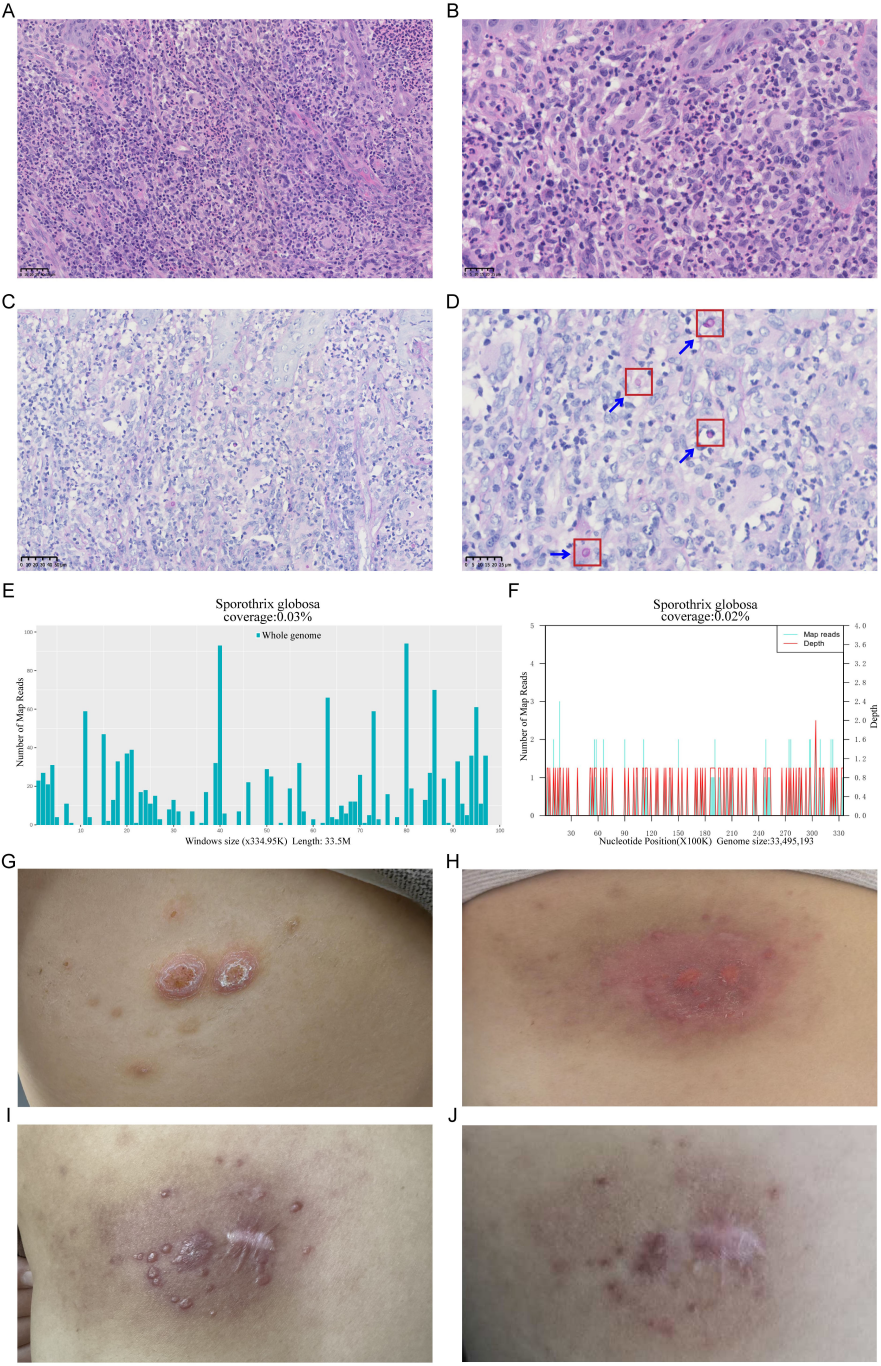
Pathogenic testing of a *Sporothrix globosa (S. globosa)*-infected skin lesion, showcasing pre- and post-treatment changes. **(A,B)** Hematoxylin-Eosin staining of the skin lesion tissue revealed numerous acute and chronic inflammatory cell infiltrates, formation of microabscesses, and scattered multinucleated giant cells, **(A)** 40×; **(B)** 80×. **(C,D)** Diastase-resistant Periodic Acid-Schiff staining of the skin lesion tissue revealed a scattered distribution of round to oval blastoconidia within the necrotic dermal tissue, **(C)** 40×; **(D)** 80×. **(E)** Distribution map of *S. globosa* detected by Metacap in skin lesion tissue. **(F)** Distribution map of *S. globosa* detected by mNGS in skin lesion tissue. **(G)** Reactive perforating collagenosis lesion on the patient’s back (2023.9.9). **(H)** *S. globosa* infection lesion on the patient’s back (2024.2.27). **(I)** Skin lesion status 20 days post-treatment. **(J)** Skin lesion status 3 months post-treatment.

On September 9, 2023, a 47 year-old female patient visited the dermatology outpatient clinic due to papules on her back. Initially, she presented with two red papules the size of a pinhead, which gradually enlarged, hardened, thickened, and developed verrucous hyperplasia. An umbilical depression appeared in the center of the lesions, with keratinized scales covering the surface. Several reddish-brown small papules were scattered around them (Figure 1G). The patient reported a 3 year history of anemia and had been treated with oral ferrous succinate tablets. Based on the patient’s symptoms and laboratory tests (Supplementary Tables 1, 2), a preliminary diagnosis of reactive perforating collagenosis was made. The patient was initially treated with intralesional injection of compound betamethasone (once a month for a total of 5 times), oral antihistamines, and topical corticosteroid ointment and asiaticoside ointment, but there was no significant improvement. On October 31, 2023, methylprednisolone (16 mg, once daily, orally for 12 days) was added to the treatment regimen, and the skin lesions gradually flattened and desquamation decreased.

In November 2023, during her treatment course, the patient self-applied a topical patch purchased online. Due to its poor air permeability and improper use, epidermal erosion developed at the affected site. Following exposure to decaying wood during an outdoor excursion, the lesion progressively exhibited redness, edema, and severe pruritus. Erythema and papules around the lesion gradually increased, merging into patches. Some areas were covered with scales, and the periphery was slightly elevated, forming a ring-like appearance (Figure 1H). Consequently, the patient visited our department again. The blood routine test results indicated that the patient had moderate anemia (Supplementary Table 3). The coagulation function tests showed that activated partial thromboplastin time 24.7 s (reference range 25–31.3 s), and the other values were in the normal range. In addition, Serum tests for HIV, hepatitis B virus, hepatitis C virus, and syphilis were negative. The three-dimensional CT scan of the skin showed no abnormalities. Clinically, skin infection is considered.

The Hematoxylin-Eosin (HE) Staining results reveal irregular hyperplasia of the epidermis, accompanied by numerous acute and chronic inflammatory cell infiltrations and fibrous scar tissue hyperplasia in the dermis. Additionally, scattered multinucleated giant cells are observed. Based on these observations, an infectious lesion is suggested (Figures 1A, B). To further identify the infectious pathogen, Diastase-resistant Periodic Acid-Schiff (DPAS) staining and MetaCAP were performed. DPAS staining revealed a scattered distribution of round to oval blastoconidia within the necrotic dermal tissue (Figures 1C, D). Meanwhile, MetaCAP detected *S. globosa* with 330 RPM reads (Reads per Million sequence reads) (Figure 1E). To further validate the results, we retested the same skin lesion tissue using mNGS, which also indicated the presence of *S. globosa* with 190 RPM reads (Figure 1F). Therefore, the patient was ultimately diagnosed with sporotrichosis caused by *S. globosa*. Treatment with itraconazole (0.2 g, twice daily) and naftifine hydrochloride and ketoconazole cream (1 g daily) was initiated. After 20 days and 3 months of follow-up, the patient’s skin rash symptoms showed significant improvement (Figures 1I, J).

## 3 Discussion

In this case, based on the clinical manifestations and laboratory tests, we made a preliminary diagnosis of reactive perforating collagenosis, although pathological examination was not conducted at that time to confirm the diagnosis definitively. The patient had a long history of anemia and the presence of skin wounds increased the risk of infection. After treatment with methylprednisolone, the skin lesions showed some improvement. However, the patient’s condition deteriorated following self-application of an online-purchased topical patch, which exhibited poor air permeability. Subsequent exposure to decaying wood further exacerbated the lesions, manifesting as progressive redness, edema, pruritus. Using MetaCAP, we detected *S. globosa* in the patient’s lesion tissue, confirming the diagnosis of infection. MetaCAP, as a high-throughput sequencing technology based on probe capture, enables rapid and accurate detection of pathogens, providing crucial diagnostic evidence for clinical practice, especially in situations where traditional culture methods have low sensitivity and are time-consuming. MetaCAP demonstrated its potential in detecting complex infections in this case, as direct detection of all pathogens within pathological tissues can reduce variability between samples and sampling times, thereby enhancing the accuracy of detection. In terms of treatment, itraconazole was selected as the first-choice medication due to its broad-spectrum antifungal activity, and the patient’s symptoms significantly improved after treatment (Figure 1). It is noteworthy that the treatment cycle for sporotrichosis is relatively long and has a significant impact on the patient’s quality of life, making prevention of infection particularly important. Through this case, we emphasize the importance of early diagnosis and personalized treatment, as well as the promising application of MetaCAP in the diagnosis of infectious diseases.

*Sporothrix* comprises a complex group, with clinically pathogenic species including *S. brasiliensis, S. schenckii, S. globosa, and S. luriei*, each possessing unique epidemiological and virulence features (1, 6, 9). *S. globosa* displays a widespread geographical distribution globally, having been successfully isolated from various continents such as North America, South America, Europe, and Asia. In Asia, *S. globosa* accounts for over 99% of all *Sporothrix* species, serving as the primary endemic species. This species has garnered extensive attention and reporting in numerous Asian countries, including China, Japan, and India. In northeast China, *S. globosa* is the most frequently reported *Sporothrix* species, exhibiting the highest incidence rate in the region, while a limited number of cases are attributed to *S. schenckii* (2, 16–18). *S. globosa* often inhabit saprotrophic environments such as soil, wood, and plants, including dead wood, peat moss, corn stalks, and hay. Both humans and animals can serve as hosts for *Sporothrix* species. Among animals, cats are the most susceptible to *Sporothrix* infections. In Asia, nearly all cat-associated cases were caused by *S. schenckii*, although there have also been reports of *S. globosa* causing cat bite wound infections (19). The primary route of transmission for sporotrichosis is through contact. In general, infections in humans and animals occur through contact with contaminated soil, wood, or plants infected with *Sporothrix* via broken skin. Therefore, farmers, gardeners, florists, and others engaged in activities such as shrub pruning, hay baling, and similar tasks are considered high-risk occupational groups for sporotrichosis. In rural northeastern China, burning wood, branches, straw, and other materials for cooking and heating increases the risk of exposure to *Sporothrix*-contaminated materials. The main method of preventing and controlling sporotrichosis is to cut off the route of transmission through contact. This can be achieved by wearing personal protective equipment such as gloves and masks, and by regularly cleaning up straw and reducing the production of decaying plants (20).

Infection is one of the significant diseases that pose a threat to human health, and rapid and accurate identification of pathogens holds great value in assisting clinical diagnosis and guiding treatment (21). Culture based methods for the diagnosis of sporotrichosis are still considered as gold standard, but this method has low sensitivity, is time-consuming, and cannot accurately identify the pathogen at the species level. There is an urgent clinical need for new diagnostic methods with shorter turnaround times than traditional cultures. With the development of molecular diagnostic technologies, metagenomic next-generation sequencing, mNGS has been widely applied in the detection of pathogenic microorganisms, but issues such as low sensitivity and high detection costs still exist (22, 23). With the aim of catering to clinical and market demands, targeted sequencing technology came into being. KingMed Diagnostics has integrated probe capture technology with next-generation sequencing technology to independently develop MetaCAP, which has been widely applied in the etiological diagnosis of various clinical infectious diseases (12–15). Compared to traditional culture method, the standout advantage of MetaCAP lies in its extensive detection range. While traditional culture method is limited to detecting only certain bacterial and fungal pathogens, MetaCAP boasts the capability to detect a wide array of microorganisms, including bacteria, fungi, viruses and parasites, across a comprehensive spectrum of sample types. It circumvents the limitation that most pathogens cannot be or are difficult to be cultured. Additionally, the probe capture-based target enrichment next-generation sequencing (NGS) excels with abilities that traditional culture method struggle to replicate, including the expertise to perform whole-genome typing, differentiate between highly homologous species, accommodate homologous viral mutations, and comprehensively cover drug-resistance sites in key pathogens of concern (24–26). In comparison to mNGS, MetaCAP stands out with its reduced data volume requirements and superior cost-effectiveness ratio (14). Specifically, the sequencing data volume needed for MetaCAP is markedly lower than that of mNGS; mNGS necessitates a qualified data volume of reads ≥10,000,000, whereas MetaCAP requires reads ≥300,000. Moreover, MetaCAP not only guarantees detection performance but also offers a more affordable price. It has the capability to simultaneously detect both DNA and RNA processes, offering a more comprehensive view of the pathogen landscape in samples. In KingMed Diagnostics, Guangzhou, Guangdong, China, a single MetaCAP test costs around 2000 RMB. Conversely, mNGS can detect either DNA or RNA pathogens individually, with a single test priced around 3,000 RMB. However, if mNGS encompasses both DNA and RNA processes, the price escalates to 5,000 RMB, and the experimental procedure becomes more time-consuming (14). Compared to mNGS and culture, the probe capture-based target enrichment NGS exhibits both superior sensitivity and a quicker detection speed. The extremely low ratio of pathogen nucleic acid to host nucleic acid results in signals being drowned out by non-informative reads, thereby constraining the sensitivity of mNGS (27). Leveraging probe capture technology, the target enrichment NGS can markedly boost detection sensitivity (25, 28). For bloodstream infections, Blood MetaCAP exhibited a significantly higher sensitivity compared to blood culture (91.3% vs. 23.2%, *P* < 0.001) and blood mNGS (91.3% vs. 69.6%, *P* = 0.001), especially for fungi and intracellular bacteria (14). Furthermore, the probe capture-based target enrichment NGS achieves a comprehensive enhancement in the scope and depth of pathogen coverage (25, 26, 28). Even in instances of complex or mixed infections, targeted enrichment demonstrates impressive performance in identifying diverse microorganisms (24, 27). In terms of detection speed, targeted enrichment can expedite the process by directly detecting and analyzing nucleic acids in specimens, outperforming culture. When compared to mNGS, targeted enrichment method’s reduced data burden facilitates a quicker detection workflow (29). On the Illumina sequencing platform, mNGS requires 10 h, whereas MetaCAP only needs 6 h on the Illumina Miniseq platform. In the context of bloodstream infections, blood MetaCAP demonstrated a faster turnaround time (16 h) compared to blood culture (50.1 ± 9.9 h) and blood mNGS (30.5 h) (14). It is worth mentioning that for pathological specimens that are difficult to re-sample, require differential diagnosis, need a clear identification of the pathogen, or need to rule out infection, the “MetaCAP + pathological morphology” fusion diagnosis brings more possibilities for precise diagnosis of infectious diseases.

The clinical treatment plans vary for different types of sporotrichosis. Itraconazole has become the first-choice medication for the treatment of lymphocutaneous sporotrichosis and cutaneous sporotrichosis due to its effectiveness, safety, and oral convenience (30, 31). The patient presented with a fixed, *S. globosa* infection on the skin of the back. Therefore, itraconazole (0.2 g, twice daily) combined with naftifine hydrochloride and ketoconazole cream (1 g daily) was selected as the primary treatment. After 20 days of follow-up, the patient’s skin lesion symptoms had alleviated and showed signs of improvement. After 3 months of follow-up, the patient’s skin lesions had basically healed, with no recurrence. It should be noted that although itraconazole is effective in treatment, it can cause various side effects and interact with over 200 other drugs, inducing adverse events such as headaches and gastrointestinal disorders. It also exhibits hepatotoxicity, teratogenicity, and embryotoxicity, and therefore cannot be used in patients with liver disease or pregnant women. For lymphocutaneous and fixed cases, potassium iodide (KI) is the preferred treatment option in many developing countries due to its high efficacy and low cost. Additionally, it can be used to treat *S. globosa* infections that are resistant to itraconazole. However, it is not recommended for disseminated extracutaneous cases, immunocompromised patients, or during pregnancy. Furthermore, adverse reactions associated with this medication, such as a metallic taste and nausea, followed by an acneiform eruption, and a complicated treatment regimen, need to be considered (32–35).

## 4 Conclusion

We report a case where the MetaCAP was successfully utilized for rapid confirmation of *S. globosa* infection, and demonstrate the remarkable efficacy of itraconazole treatment. Early diagnosis is crucial for personalized treatment, prevention of disease progression, reduction of complication risks, optimization of treatment outcomes, and improvement of patient prognosis. With its high efficiency and precise pathogen detection capabilities, MetaCAP provides strong support for early diagnosis and personalized treatment of infectious disease.

## Data Availability

The original contributions presented in this study are included in this article/Supplementary material, further inquiries can be directed to the corresponding author.
