# Supplementary material for: A case report of rapid diagnosis of Sporothrix globosa infection using MetaCAP
Source: Front Med (Lausanne). 2025 Aug 11;12:1644400. doi: 10.3389/fmed.2025.1644400 (PMC12375437; doi:10.3389/fmed.2025.1644400)
Supplement: Supplementary file 1 [file Table_1.docx]

Supplementary Material

**Supplementary Table 1：2023-9-9 Blood Routine Report**

| ****Test Item**** | ****Result**** | ****Reference Range**** | ****Unit**** |
| --- | --- | --- | --- |
| white blood cell | 6.5 | 3.5 ~ 9.5 | 10^9^/L |
| neutrophil | 68.2 | 40 ~ 75 | % |
| lymphocyte | 24.2 | 20 ~ 50 | % |
| red blood cell | 4.21 | 3.8 ~ 5.1 | 10^12^/L |
| hemoglobin | 73⬇ | 115 ~ 150 | g/L |
| hematocrit | 27.5⬇ | 35 ~ 45 | % |
| mean corpuscular volume | 65.3⬇ | 82 ~ 100 | fl |
| mean corpuscular hemoglobin | 17.3⬇ | 27 ~ 34 | pg |
| mean corpuscular hemoglobin concentration | 265⬇ | 316 ~ 354 | g/L |
| red blood cell distribution width | 18.4⬆ | 10.6 ~ 15 | % |
| platelet | 273 | 100 ~ 300 | 10^9^/L |

**Supplementary Table 2: 2023-9-9 Serum biochemistry tests**

| ****Test Item**** | ****Result**** | ****Reference Range**** | ****Unit**** |
| --- | --- | --- | --- |
| [glucose](javascript:;) | 6.83⬆ | 3.6 to 6.1 | mmol/L |
| [total](javascript:;) [cholesterol](javascript:;) | 3.81 | 3.10 ~ 5.70 | mmol/L |
| [triglyceride](javascript:;) | 0.89 | 0.56~ 1.70 | mmol/L |
| aspartate aminotransferase | 20 | 0 ~ 40 | U/L |
| alanine aminotransferase | 13 | 0 ~ 35 | U/L |
| glutamyl transpeptidase | 11 | 0 ~ 40 | U/L |
| alkaline phosphatase, | 46 | 42 ~ 128 | U/L |
| total protein | 72.5 | 63.0 ~ 82.0 | g/L |
| albumin | 46.5 | 35.0 ~ 50.0 | g/L |
| [unconjugated](javascript:;) [bilirubin](javascript:;) | 4.6 | 0.0 ~ 19.0 | umol/L |
| conjugated bilirubin | 0.0 | 0.0 ~ 5.0 | umol/L |
| urea | 5.59 | 2.50 ~ 6.10 | mmol/L |
| creatine | 56 | 44 ~ 106 | umol/L |
| uric acid | 178 | 149 ~ 446 | umol/L |
| lactate dehydrogenase | 131 | 120 ~ 246 | U/L |
| creatine kinase | 48 | 30 ~ 170 | U/L |

**Supplementary Table 3：The Blood Routine Report on admission**

| ****Test Item**** | ****Result**** | ****Reference Range**** | ****Unit**** |
| --- | --- | --- | --- |
| white blood cells | 7.8 | 3.5 ~ 9.5 | 10^9^/L |
| neutrophils | 67.6 | 40 ~ 75 | % |
| lymphocytes | 24 | 20 ~ 50 | % |
| red blood cells | 4.66 | 3.8 ~ 5.1 | 10^12^/L |
| hemoglobin | 78 ⬇ | 115 ~ 150 | g/L |
| hematocrit | 28.5⬇ | 35 ~ 45 | % |
| mean corpuscular volume | 61.2⬇ | 82 ~ 100 | fl |
| mean corpuscular hemoglobin | 16.8⬇ | 27 ~ 34 | pg |
| mean corpuscular hemoglobin concentration | 274⬇ | 316 ~ 354 | g/L |
| red blood cell distribution width | 19⬆ | 10.6 ~ 15 | % |
| reticulocyte percentage | 2.6⬆ | 0.5 ~ 1.5 | % |
| reticulocyte count | 122.1⬆ | 24 ~ 84 | 10^9^/L |
| immature reticulocyte fraction | 17.6⬆ | 2.1 ~ 13.8 | % |
| reticulocyte-hemoglobin | 18⬇ | 28 ~ 35 | pg |
| platelets | 310⬆ | 100 ~ 300 | 10^9^/L |
