# Supplementary material for: A case report of rapid diagnosis of Sporothrix globosa infection using MetaCAP
Source: Front Med (Lausanne). 2025 Aug 11;12:1644400. doi: 10.3389/fmed.2025.1644400 (PMC12375437; doi:10.3389/fmed.2025.1644400)
Supplement: Supplementary file 2 [file Table_2.pdf]

CARE Checklist of information to include when writing a case report

Topic	Item	Checklist item description	Reported on Line
Title	1	The diagnosis or intervention of primary focus followed by the words “case report”	Rapid diagnosis of *Sporothrix globosa* infection using MetaCAP: a case report
Key Words	2	2 to 5 key words that identify diagnoses or interventions in this case report, including "case report"	*Sporothrix globosa*, MetaCAP, mNGS, Itraconazole,Case report
Abstract (no references)	3a	Introduction: What is unique about this case and what does it add to the scientific literature?	*Sporothrix globosa (S. globosa)* is a significant pathogenic fungus responsible for causing sporotrichosis. Metagenomics Capture (MetaCAP), a high-throughput sequencing technology for pathogen nucleic acid detection based on probe capture, facilitates early diagnosis of *S. globosa* infections.
	3b	Main symptoms and/or important clinical findings	A 47-year-old female, initially diagnosed with reactive perforating collagenosis, developed epidermal erosion at the affected site after self-applying a poorly air-permeable topical patch purchased online. She subsequently exhibited progressive redness, edema, severe pruritus, and an increase in papules that coalesced into plaques following exposure to decaying wood.
	3c	The main diagnoses, therapeutic interventions, and outcomes	The diagnosis of sporotrichosis caused by *S. globosa* was confirmed through a pathological examination of the affected skin tissue and MetaCAP testing. Then，She was treated with itraconazole and naftifine-ketoconazole cream. After a three-month follow-up, the patient's skin rash showed significant improvement.
	3d	Conclusion—What is the main “take-away” lesson(s) from this case?	we emphasize the importance of early diagnosis and personalized treatment, as well as the promising application of MetaCAP in the diagnosis of infectious diseases.
Introduction	4	One or two paragraphs summarizing why this case is unique (may include references)	*S. globosa* primarily affects the skin but can also involve mucous membranes, subcutaneous tissues, and adjacent lymphatic vessels. In severe cases, it can disseminate through the blood and lymphatic system, causing systemic damage and posing a life-threatening risk. Prompt

			diagnosis and treatment of *S. globosa* infections are crucial to prevent disease progression. MetaCAP is a high-throughput sequencing technology for pathogen nucleic acid detection based on probe capture, which allows for direct detection and analysis of the genetic material of all microorganisms in various types of clinical samples. It significantly shortens the diagnosis time and provides crucial information for the diagnosis and treatment of the disease.
Patient Information	5a	De-identified patient specific information	A 47-year-old female patient
	5b	Primary concerns and symptoms of the patient	Papules on her back
	5c	Medical, family, and psycho-social history including relevant genetic information	A three-year history of anemia
	5d	Relevant past interventions with outcomes	The patient had been treated with oral ferrous succinate tablets.<br>The blood routine test results on admission indicated that the patient had moderate anemia.
Clinical Findings	6	Describe significant physical examination (PE) and important clinical findings	The skin lesion gradually became redness, edema, and severe pruritus. Erythema and papules around the lesion gradually increased, merging into patches. Some areas were covered with scales, and the periphery was slightly elevated, forming a ring-like appearance.
Timeline	7	Historical and current information from this episode of care organized as a timeline	On September 9, 2023: The patient was admitted to the hospital due to back papules and was initially diagnosed with reactive perforating collagenosis. Treatment was initiated with compound betamethasone, antihistamines, as well as topical corticosteroid ointment and asiaticoside ointment.<br>On October 31, 2023: As the patient's symptoms showed no improvement, methylprednisolone was added to the treatment regimen. Subsequently, the skin lesions gradually flattened, and desquamation decreased.<br>In November 2023: The skin lesions developed epidermal erosion after self-applying a poorly air-permeable topical patch purchased online. The patient subsequently exhibited progressive redness, edema, severe pruritus, and an increase in papules that coalesced into

			plaques following exposure to decaying wood. Through pathological examination and MetaCAP testing, the patient was diagnosed with sporotrichosis caused by *S. globosa.* Treatment was commenced with itraconazole, naftifine hydrochloride and ketoconazole cream.<br>After 20 days and 3 months of follow-up, the patient's skin rash symptoms demonstrated significant improvement.
Dignostic Assessment	8a	Diagnostic testing (such as PE, laboratory testing, imaging, surveys)	Blood Routine Test, Hematoxylin-Eosin Staining, Diastase resistant Periodic Acid-Schiff staining, MetaCAP, mNGS.
	8b	Diagnostic challenges (such as access to testing, financial, or cultural)	The specific type of infectious pathogen cannot be determined through pathology. The patient's skin lesion tissue has been processed into paraffin - embedded sections, rendering it unsuitable for further culture.<br>Re - sampling would increase the patient's suffering, prolong the diagnostic duration, and there is a certain degree of variability among different samples.
	8c	Diagnosis (including other diagnoses considered)	Sporotrichosis caused by *S. globosa*.
	8d	Prognosis (such as staging in oncology) where applicable	Significant improvement
Therapeutic Intervention	9a	Types of therapeutic intervention (such as pharmacologic, surgical, preventive, self-care)	Pharmacologic
	9b	Administration of therapeutic intervention (such as dosage, strength, duration)	Itraconazole (0.2g, twice daily)<br>Naftifine hydrochloride and ketoconazole cream (1g daily)
	9c	Changes in therapeutic intervention (with rationale)	/
Follow-up and Outcomes	10a	Clinician and patient-assessed outcomes (if available)	Significant improvement
	10b	Important follow-up diagnostic and other test	Monitor the skin lesions on the patient's back and take photos for record.

		results	After 20 days and 3 months of follow-up, the patient's skin rash symptoms showed significant improvement.
	10c	Intervention adherence and tolerability (How was this assessed?)	Good; Assessed via methods like patient self-reports, pill counts, telephone follow-ups to gauge adherence; tolerability assessed by monitoring adverse events, side effects, and patient feedback.
	10d	Adverse and unanticipated events	/
Discussion	11a	A scientific discussion of the strengths AND limitations associated with this case report	A case of *S. globosa* infection was promptly diagnosed through MetaCAP and effectively treated with itraconazole.<br>A drawback of this case report is the small sample size. Further studies with a larger number of cases are required to validate the utility of metacap in diagnosing infectious diseases.
	11b	Discussion of the relevant medical literature with references	Refer to the manuscript
	11c	The scientific rationale for any conclusions (including assessment of possible causes)	The basis for our conclusions encompasses our deliberations on this case as well as our study of previously published literature.
	11d	The primary "take-away" lessons of this case report (without references) in a one paragraph conclusion	We report a case where the MetaCAP was successfully utilized for rapid confirmation of *S. globosa* infection, and demonstrate the remarkable efficacy of itraconazole treatment. Early diagnosis is crucial for personalized treatment, prevention of disease progression, reduction of complication risks, optimization of treatment outcomes, and improvement of patient prognosis. With its high efficiency and precise pathogen detection capabilities, MetaCAP provides strong support for early diagnosis and personalized treatment of infectious disease.
Patient Perspective	12	The patient should share their perspective in one to two paragraphs on the treatment(s) they received	I'm so pleased with the treatment. Metacap quickly located the infection's culprit, giving the doctor a clear path. The treatment plan they came up with worked wonders. My symptoms improved rapidly, and I'm on the road to recovery. I can't thank the medical staff enough for their skill and the use of this helpful technology.
Informed Consent	13	Did the patient give informed consent? Please provide if requested	Yes ☑ No ☐
